# Hunter New England Area Health Service pilot telehealth high risk foot clinic: connecting Tamworth and Newcastle

**DOI:** 10.1186/1757-1146-4-S1-P33

**Published:** 2011-05-20

**Authors:** Nicole Martin, Chris Perfrement, Kate Connell, Bronwyn Hardy, Louise Maye

**Affiliations:** 1 Podiatry and Footcare Services, Hunter New England Area Health Service, NSW Health, Newcastle, NSW, 2300, Australia; 2 Podiatry Services, Hunter New England Area Health Service, NSW Health, Tamworth, NSW, 2340, Australia

## Background

Clients across Hunter New England Area Health Service (HNEAHS) with complex diabetes foot complications traditionally had to travel to Newcastle to access multidisciplinary high risk foot services; with some travelling up to five hours each way to attend the High Risk Foot Clinic in Newcastle. Ideally rural clients should be able to access multidisciplinary high risk foot services close to their home.

## Methods

A six-month pilot Telehealth High Risk Foot Clinic was established between Newcastle and Tamworth, with clinicians in Newcastle providing Telehealth multidisciplinary input for clients attending the Tamworth Podiatry Clinic.

## Results

Over the six month pilot phase (February – August 2010) a total of eight clients were seen through the HNEAHS Telehealth High Risk Foot Clinic; seven presented with foot wounds and one presented with an acute Charcot Neuroarthropathy. Two clients (25%) refused gold standard treatment; one was subsequently discharged from the High Risk Foot Clinic and the other did not return for follow-up treatment. Of the remaining six clients, four (75%) achieved successful resolution of their presenting complaint. Clients surveyed in the evaluation phase indicated that they would prefer to attend the Telehealth clinic at Tamworth as it is closer to home, meaning less travel time and expense as well as reducing the impact on their work and/or family commitments. None of the clients felt unsatisfied in any way about the Telehealth model and all felt very satisfied by the impact that the clinic made on their quality of life. A cost analysis of the pilot model showed that this type of service is of cost benefit and has the potential to prevent lower limb amputations in high risk clients residing in rural areas.

## Conclusions

The pilot clinic resulted in many positive benefits to high risk foot clients living in rural areas. Good clinical outcomes were achieved, the client’s quality of life was improved and cost benefits were attained. Also of note, were the strong relationships that were formed between Newcastle and Tamworth; resulting in rural clinicians being empowered with knowledge of the management of the high risk foot.

